# Engineering cytoplasmic acetyl-CoA synthesis decouples lipid production from nitrogen starvation in the oleaginous yeast *Rhodosporidium azoricum*

**DOI:** 10.1186/s12934-019-1250-6

**Published:** 2019-11-14

**Authors:** Silvia Donzella, Daniela Cucchetti, Claudia Capusoni, Aurora Rizzi, Silvia Galafassi, Gambaro Chiara, Concetta Compagno

**Affiliations:** 1 Department of Food, Environmental and Nutritional Sciences, University of Milan, Milan, Italy; 2 Versalis SPA, Green Chemistry CRNO, Novara, Italy; 3 Eni S.p.A.—Renewable Energy and Environmental R&D Center—Istituto Eni Donegani, Novara, Italy; 4 Water Research Institute, National Research Council, Verbania, Italy

**Keywords:** *Rhodosporidium azoricum*, Lipid production, Oleaginous yeasts, Phosphoketolases, Phosphotransacetylase, Lignocellulosic hydrolysates, Renewable resources

## Abstract

**Background:**

Oleaginous yeasts are able to accumulate very high levels of neutral lipids especially under condition of excess of carbon and nitrogen limitation (medium with high C/N ratio). This makes necessary the use of two-steps processes in order to achieve high level of biomass and lipid. To simplify the process, the decoupling of lipid synthesis from nitrogen starvation, by establishing a cytosolic acetyl-CoA formation pathway alternative to the one catalysed by ATP-citrate lyase, can be useful.

**Results:**

In this work, we introduced a new cytoplasmic route for acetyl-CoA (AcCoA) formation in *Rhodosporidium azoricum* by overexpressing genes encoding for homologous phosphoketolase (Xfpk) and heterologous phosphotransacetylase (Pta). The engineered strain PTAPK4 exhibits higher lipid content and produces higher lipid concentration than the wild type strain when it was cultivated in media containing different C/N ratios. In a bioreactor process performed on glucose/xylose mixture, to simulate an industrial process for lipid production from lignocellulosic materials, we obtained an increase of 89% in final lipid concentration by the engineered strain in comparison to the wild type. This indicates that the transformed strain can produce higher cellular biomass with a high lipid content than the wild type. The transformed strain furthermore evidenced the advantage over the wild type in performing this process, being the lipid yields 0.13 and 0.05, respectively.

**Conclusion:**

Our results show that the overexpression of homologous Xfpk and heterologous Pta activities in *R. azoricum* creates a new cytosolic AcCoA supply that decouples lipid production from nitrogen starvation. This metabolic modification allows improving lipid production in cultural conditions that can be suitable for the development of industrial bioprocesses using lignocellulosic hydrolysates.

## Background

Production of biofuels from renewable resources has been receiving growing attention due to global energy demand and environmental concerns. Among the investigated microorganisms, oleaginous yeasts are able to accumulate very high levels of neutral lipids, in some species up to 70% of their dry mass, under particular culture conditions, providing a promising strategy to reducing our dependence on fossil fuels. Nutrient limitations such as nitrogen, phosphate or sulphate are determining parameters for lipid accumulation [1–3], as well as pH and aeration rate are other important parameters influencing lipid production [4–6]. The C/N ratio of the medium is a crucial parameter. In oleaginous yeast in fact, upon nitrogen limitation, carbon is diverted to lipid synthesis. ATP citrate lyase (ACL) is the enzyme that catalyzes the conversion of citrate into acetyl-CoA and oxaloacetate. Citrate accumulation and transportation into the cytoplasm is triggered by inhibition of isocitrate dehydrogenase (ICDH), the enzyme responsible for the conversion of isocitrate to oxoglutarate in the TCA cycle [7]. This inhibition is dependent on reduced cellular AMP content, which results from activation of a nitrogen-scavenging enzyme identified as AMP deaminase. This enzyme deaminates adenosine monophosphate (AMP), thus freeing ammonia, which can be utilized by the cell as a nitrogen source. This regulatory mechanism makes necessary the use of two-steps processes in order to achieve high biomass and lipid production [8], requiring a growth phase (with nitrogen present) and a lipid production phase (with nitrogen depleted), which prolongs the time of fermentation.

Recently, extensive efforts have been made to engineer various microorganisms as lipid bio- factory [9–11]. Work using “flux push-and-pull” strategies in *Yarrowia lipolytica* has led to the construction of efficient single-cell oil factories [12–14]. In the oleaginous yeast *Rhodosporidium toruloides*, which is a robust lipid producer, multi-omics information and established reliable genetic manipulation strategies have been obtained [15, 16]. With the aim to simplify the fermentation process, the decoupling of lipid production from nitrogen depletion, by establishing a cytosolic AcCoA formation pathway alternative to the one catalysed by ACL, can be then useful. In that regard, approaches of metabolic engineering have been explored introducing new pathways, as from pyruvate in *Y. lipolytica* [17] and in *Trichosporon oleaginosus* [18]. In this context, interesting enzymes are the phosphoketolases (Xfpk, EC 4.1.2.9 and EC 4.1.2.22), typical enzymes of heterofermentative and facultatively homofermentative lactic acid bacteria. These enzymes cleave the pentose phosphate pathway (PPP) intermediate xylulose-5-phosphate, and/or the glycolytic intermediate fructose-6-phosphate, producing acetyl-phosphate (AcP) and glyceraldehyde-3-phosphate or erythrose-4-phosphate, that can be recycled in glycolysis and in the PPP, respectively [19]. AcP, in turn, can be converted to acetyl-CoA (AcCoA) by phosphotransacetylase (Pta; EC 2.3.1.8), without requiring ATP. This metabolic pathway results attractive for targeting production of secondary metabolites that are derived from AcCoA as precursor, representing a synthetic route that is energy and carbon neutral, with the highest theoretical yield [20–23]. Heterologous Xfpk and Pta encoding genes have been expressed in *Y. lipolytica* [17]. Recently, a bacterial Pta has been expressed in *R. toruloides*, based on the assumption that a gene encoding Xfpk, for potential formation of AcP, is present in its genome [15, 24].

**Fig. 1:**
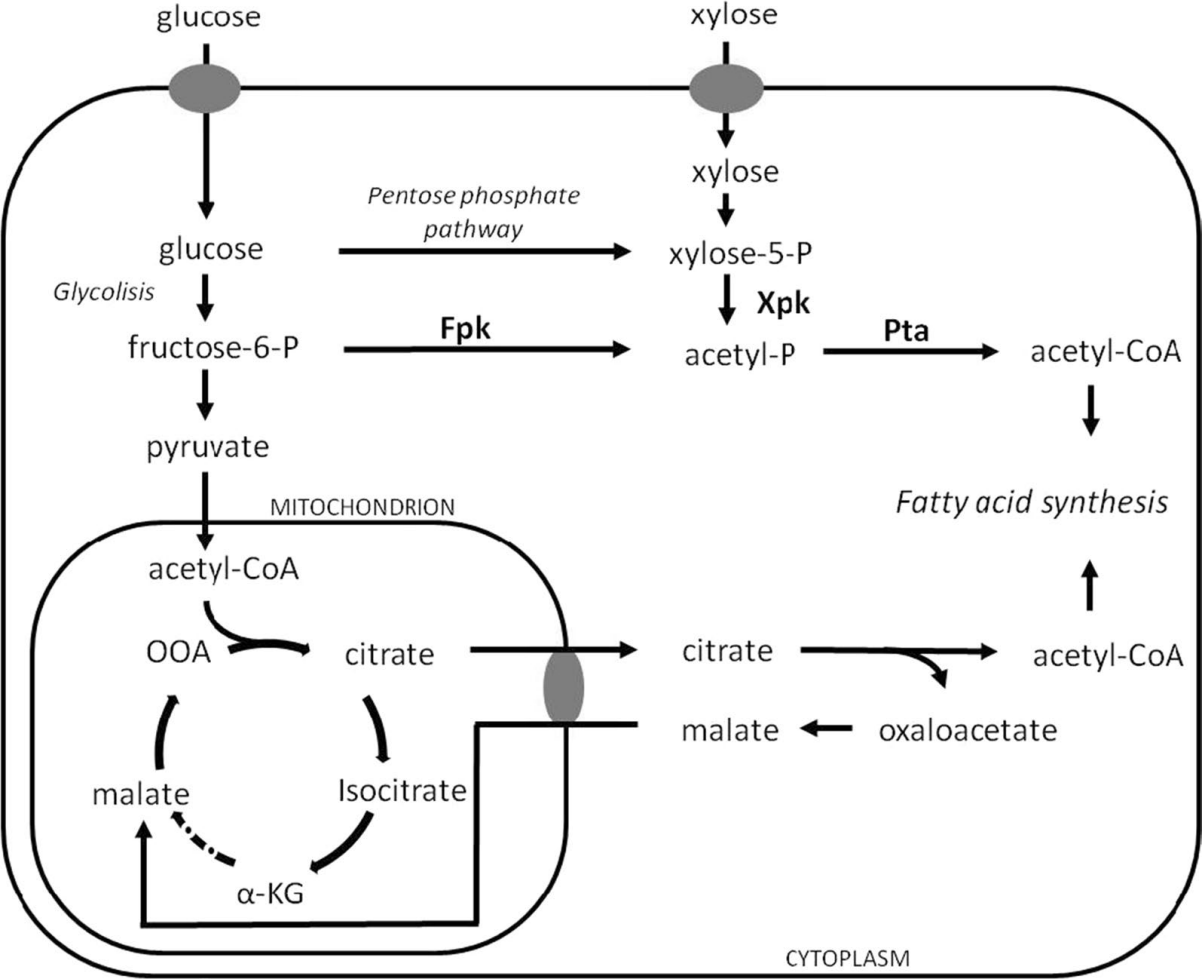
Pathways involved in AcCoA synthesis. In bold the introduced enzymes

In this work, we adopted a similar metabolic strategy in the oleaginous yeast *Rhodosporidium azoricum*. Previous studies demonstrated that this species possess characteristics suitable for the development of industrial lipid production, like a wide range of carbon sources utilization, tolerance to inhibitors, and lipid accumulation up to 60% [5, 25]. We identified and overexpressed the homologous gene encoding for a putative Xfpk, together with the heterologous gene from *Bacillus subtilis* encoding for Pta (Fig. 1). By this approach, we obtained transformed strains that showed higher lipid content. In particular, the PTAPK4 strain reached a higher lipid production in comparison to the wild type strain even in presence of nitrogen in the medium, demonstrating that the metabolic manipulation allowed the decoupling of lipogenesis from nitrogen starvation.

## Methods

### Strains

The yeast strains used in this work are DBVPG 4620 reclassified as *R. azoricum* [5] and the mutant *ura5* obtained as described below. For long-term storage, yeast strains were maintained at − 80 °C on 15% (v/v) glycerol and 85% (v/v) YPD media (10 g/l yeast extract, 20 g/l peptone and 20 g/l glucose). *Escherichia coli* strain used for cloning and plasmid propagation was One Shot^®^ TOP10. For long-term storage, yeasts and bacteria strains were maintained at − 80 °C on 15% (v/v) glycerol and 85% (v/v) respectively.

### Media composition

Medium YPD was composed of 20 g/l glucose, 20 g/l peptone, 10 g/l yeast extract. Medium B contained glucose or xylose at different concentrations (20 or 50 g/l), KH_2_PO_4_ 1 g/l, MgSO_4_ 7H_2_O 0.05 g/l, NaCl 0.01 g/l, CaCl_2_ 0.01 g/l, yeast extract 1 g/l and (NH_4_)_2_SO_4_ at different concentration in order to obtain several C/N ratios. C/N ratio was calculated based on molar concentration of the single components. In particular we called B75 the medium B containing (NH_4_)_2_SO_4_ 1 g/l (0.21 g N), and yeast extract 1 g/l (containing 0.1 g N, according to supplier specification), that gives a total amount of 0.022 N moles, corresponding to a final molar C/N ratio of 75.3. B37 and B20 medium had the same composition as B75 except a higher (NH_4_)_2_SO_4_ concentration (2.5 g/l and 5 g/l respectively). Medium YNB contained yeast nitrogen base 0.17 g/l (Difco, BD, Milan, Italy), glucose 20 g/l, agar 20 g/l. All media were buffered with 0.1 M MES (2-(*N*-morpholino) ethane-sulfonic acid (Sigma Aldrich, Milan, Italy) to maintain pH 6.

For fed-batch cultivations the medium contained glucose 33.3 g/l, xylose 16.7 g/l, (NH_4_)_2_SO_4_ 5 g/l, corn steep solids (Sigma) 5 g/l, MgSO_4_ 7H_2_O 0.3 g/l, CaCl_2_ 2H_2_O 0.06 g/l, KH_2_PO_4_ 6 g/l, yeast extract 2 g/l.

*Escherichia coli* strains used for cloning and plasmid propagation were cultured in LB medium (5 g/l yeast extract, 10 g/l peptone, 10 g/l NaCl) containing 100 mg/ml ampicillin.

### Shake-flask cultivation

Baffled flasks were filled with medium at liquid–air ratio of 1:5, and were incubated in a rotary shaker at 200 rpm, at 30 °C. As seed culture, strains were cultured to exponential growth phase on YPD medium. Cultures on different media were inoculated at a concentration of 0.1 OD_660_ from seed cultures. Flask cultivations were performed in triplicate.

### Selection of *ura5* mutant

In order to obtain the DBVPG 4620 *ura5* mutant, 1 OD_660_ of cells were plated on YNB supplemented with uracil 50 mg/l in presence of 5-fluoroorotic acid hydrate 0.1 g/l. Plates were irradiated with UV light for 30 s and incubated until colonies formation. To confirm the auxotrophy for uracil, *RaURA5* gene was sequenced and compared to the wild type sequence, in order to identify DNA mutations.

### DNA extraction

To isolate genomic DNA, pellets of 30 OD_660_ of cells were resuspended in 0.5 ml of 0.05 M Tris–HCl/0.02 M EDTA at pH 7.5. This suspension was transferred to a precooled tube with an equal volume of glass beats (425–600 µm). Mechanical lysis was performed using a TissueLyser LT alternating 2 min of agitation at 50 Hertz with 1 min in ice for 4 cycles. The supernatant was added with 25 μl of SDS 20% (w/v) and processed according to Querol et al. [26].

### Assembly of pTU-PT-PTAPK vector

All primers employed for plasmid assembly are listed in Additional file 1: Table S1. Molecular biology reagents were purchased from ThermoFisher. Phusion High-Fidelity DNA Polimerase was used for PCR product amplification, and restriction enzymes were used for vector assembly. NucleoSpin^®^Plasmid, NucleoSnap^®^ Plasmid Midi and PCR clean-up was purchased by Macherey–Nagel.

For the construction of the expression vector pTU-PT-PTAPK, pCR^®^II TOPO^®^ Invitrogen was initially employed. For the assembly of specific expression system for *R. azoricum*, the expression cassette pTU-PT composed by *R. azoricum PGK* promoter, multiple cloning site and *Ashbya gossypii TEF1* terminator was cloned in pCR^®^II TOPO^®^ Invitrogen using the primers reported in Additional file 1: Table S1. *PGK* promoter was amplified from genomic DNA of *R. azoricum* (primers P_PGK_f and P_PGK_r) and *TEF1* terminator (primers T_TEF_f and T_TEF_r) was amplified from pYC030 plasmid. Selective marker used for transformation was *RaURA5* gene, amplified from genomic DNA (primers Uraf_plus and Urar_plus) of *R. azoricum*, digested with BamHI and cloned in the expression system.

The *Pta* sequence encoding for phosphotransacetylase of *B. subtilis* (EC 2.3.1.8) was optimized for expression in *R. azoricum* and synthetized by Baseclear. The synthetic gene was amplified from pUC57-*Bspta* plasmid (primers ForPTA and RevPTA), digested with NdeI and cloned in pTU-PT vector, obtaining pTU-PT-PTA plasmid.

**Fig. 2:**
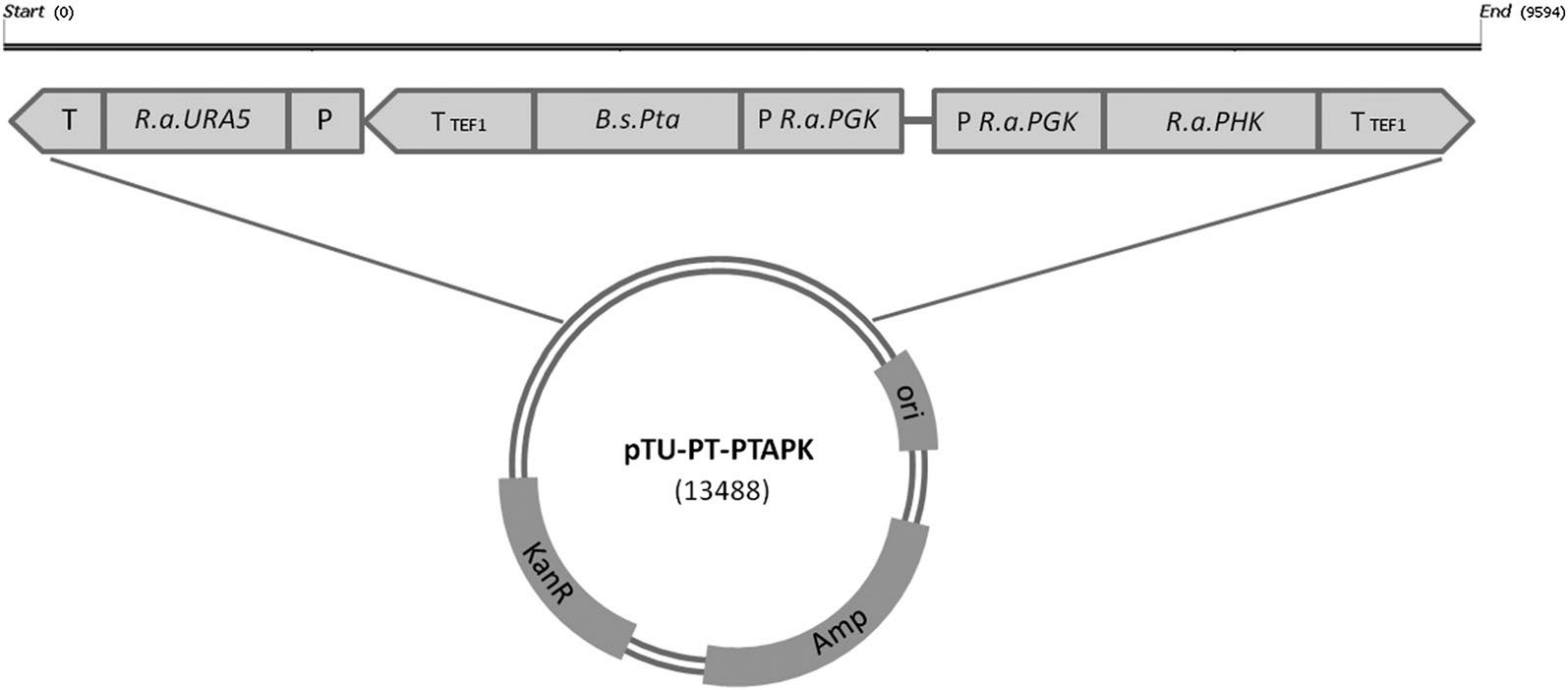
Structure of the cassette and plasmid for *BsPta* and *RaPHK* expression

The sequence encoding for phosphoketolase (*RaPHK*) was amplified from cDNA of *R. azoricum,* digested with NdeI and cloned in pTU-PT system, obtaining pTU-PT-PK plasmid. The expression cassette PT-PK vas amplified (PkX_for2 and PkX_rev2) from pTU-PT-PK plasmid digested with XbaI and cloned in pTU-PT-PTA plasmid. The plasmid pTU-PT-PTAPK (Fig. 2) was linearized with PciI and used to transform *R. azoricum*.

### Trasformation protocol

To transform *R. azoricum ura5* strain we employed the LiAc/DMSO protocol as described in Hill et al. [27]. Briefly, cells were harvested in early exponential phase, washed in LiAc/TE and resuspended in order to have 1 × 10^9^ cells/ml. At 100 µl of this solution was added 10–20 µg of linearized vector. PEG4000 solution 50% (w/v) PEG4000 in LiAc solution is added, the contents mixed by inverting 4–6 times, then the tube is placed at 30 °C for 45 min. DMSO is added to give an approximate 10% (v/v) DMSO solution in the tube. The content was mixed by inversion then heat shocked at 42 °C for 5 min. Cells are spun at 12,000*g* just long enough to pellet (usually 2 min), washed in distilled water, spun for 2 min, then resuspended in 1 ml of distilled water. Transformed cells (10^8^ cells) were plated on YPD media and scored after 3 days of growth at 30 °C.

### mRNA quantification

Cells were harvested after 24 h of shake flask growth in B20 medium at 30 °C, pellets of 30 OD_660_ of cells were frozen rapidly by submersing in liquid nitrogen and stored at – 80 °C. Total mRNA was then extracted using Presto^TM^ Mini RNA Yeast Kit (Geneaid, RBY050) and treated with DNase I (Sigma-Aldrich) to remove genomic DNA. CDNA was synthesized from mRNA using QuantiTect Reverse Transcription Kit (Qiagen). RT-qPCR was performed using QPCR Green Master Mix LRox (biotechrabbit) in a CFX96 Real-Time System C1000 thermocycler (Bio-Rad). Specific primers are listed in Additional file 1: Table S1. Standard curves for each couple of primers were generated using serial dilutions of genomic DNA. The α-tubulin gene (AT, XM_016421364.1) was used as reference gene. Cycling parameters were: (i) 95 °C for 3 min, (ii) repeated 95 °C for 15 s, 61 °C for 30 s for 37 times, and (iii) for dissociation curve, 95 °C for 6 s. All data points were collected from three biological replicates and analyzed by Bio-Rad CFX Manager software.

### Fed batch cultivation

Fed-batch cultures were performed in a 1 l bioreactor (Sartorius Stedim), with a starting volume of 800 ml. Temperature was set at 30 °C and the air inlet at 1 vol/vol/min.

Dissolved oxygen concentration was measured by Hamilton polarographic oxygen probe and was automatically kept above 30% of saturation by stirring cascade (500–1050 rpm). Foam formation was controlled by the automatic addition of a silicon antifoaming agent (Sigma 204) controlled by a foam probe. The pH, measured by Mettler Toledo pH electrode, was automatically adjusted to 5.0 by adding 2.5 M KOH. After 24 h of cultivation additional yeast extract at 2 g/l was supplemented. (NH_4_)_2_SO_4_ was feeded, up to 46 h of cultivation, from a 254.4 g/l solution to maintain a low C/N ratio (20–30 molar ratio). Carbon sources were added from a 420 g/l solution (glucose 294 g/l and xylose 126 g/l), during the process, in order to keep total concentration above 30 g/l (feed flow rate was varied according to the glucose consumption rate and ranged from 3.5 to 12 ml h^−1^).

### Cell growth and dry weight determination

Samples were collected from flasks and bioreactors at appropriate intervals to monitor cell growth by measuring the optical density at 660 nm with a spectrophotometer, after appropriate dilution. For dry weight determination, culture samples were filtered on a 0.45 μm glass microfiber GF/A filter (Whatman), washed and dried for 24 h at 80 °C.

### Sugar and ammonium assays

Samples were collected from flasks and bioreactors at appropriate intervals, supernatants (stored at − 20 °C) were obtained by centrifugation and used to assay extracellular components. Glucose, ammonium and xylose concentration were determined by commercial enzymatic kits (Roche, cat. No. 1 0716251, Roche, cat. No. 1 1112732 035, Megazyme, cat. No. K-XYLOSE, respectively). All the assays were performed in triplicate and standard deviations varied between 1 and 5%.

The biomass and product yields were calculated as the ratio between the total amount of biomass/products and the total amount of consumed sugar. Specific glucose and ammonium consumption rates have been calculated according to van Hoek et al. [28].

### Lipid assay

Lipid content was determined by the sulfo-phospho-vanilline colorimetric method (Spinreact) on washed cell pellets, suspended in 0.5 ml of cold redistilled water. The assays were performed in triplicate and standard deviations varied between 1 and 5%.

## Results and discussion

### Engineering acetyl-CoA synthesis in *R. azoricum* by overexpression of homologous Xfpk and introduction of heterologous Pta activities

The genome of *R. azoricum* DBVPG4620 has been sequenced (unpublished data). Genomic sequences encoding for *RaURA5* and *RaPHK* genes were identified based on homology with the corresponding genes in *R. toruloides* obtained from NCBI database (https://www.ncbi.nlm.nih.gov/). The deduced amino acid sequences showed similarity of 84% and 75% with the proteins corresponding to orotatephosphoribosyltransferase and phosphoketolase (Xfpk), respectively. The native promoter of the gene encoding for phosphoglycerate kinase (*RaPGK*) was identified and used to obtain a strong and constitutive expression of the *RaPHK* gene, based on the reported observations about the use of this promoter in *R. toruloides* [16] (Fig. 2). The same strategy was applied to enable expression of the heterologous codon optimized gene *Pta* from *B. subtilis* (Fig. 2). We developed a transformation protocol for *R. azoricum* based on the complementation of uracil auxotrophy of the *ura5* mutant (Additional file 1: Fig. S1), by using the homologous *RaURA5* gene as selective marker (Fig. 2). The recombinant cassette was randomly integrated in the *R. azoricum* genome by transformation, obtaining several clones that were analysed in order to verify the integration of the gene cassettes (Additional file 1: Fig. S2). Among positively transformed clones, upon cultivation under lipogenic conditions on medium B at high C/N ratio (C/N 75), only one clone showed a lower lipid production than the host *ura5* strain (control strain), having the others similar or higher levels (Additional file 1: Fig. S3). This could be expected, based on locus effect caused by random integration of the gene cassette into the *R. azoricum* genome, which can affect biomass as well as lipid production. However, as observed for other engineered strains [16, 24], the analysis of integration locus could provide no conclusive information about putative effects on lipid production, due to the fact that integration can occur in locus encoding proteins of unknown effect on biomass/lipid metabolism, or even hypothetical proteins. This problem will be overcome when strategies for homologous integration into specific locus will be available.

**Fig. 3:**
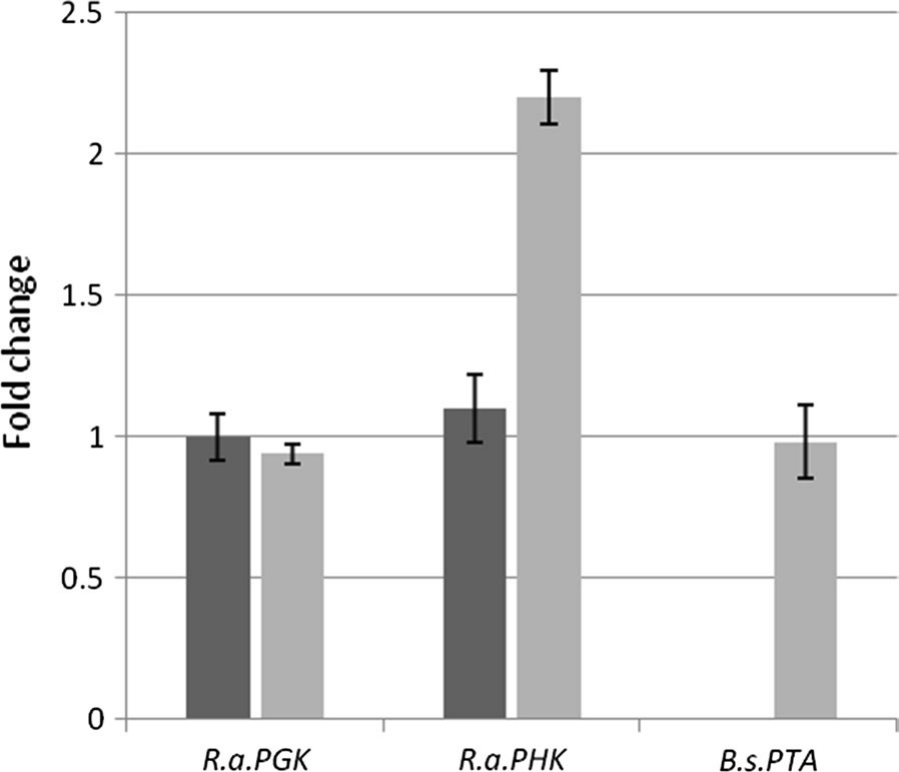
mRNA expression level of *RaPGK*, *RaPHK* and *BsPta* in the *ura5* control strain (dark gray bars) and in the PTAPK4 strain (light gray bars)

In order to check the expression of the cloned genes, quantitative RT-PCR was performed in the best performing strain PTAPK4. Due to the choice of employing the *RaPGK* promoter for the expression of both genes, we analysed also the expression of *RaPGK* to obtain information about its strength and putative locus effects on the expression of inserted genes. No difference in the expression level of *RaPGK* was found for *ura5* and PTAPK4 genetic background (Fig. 3). In *ura5* strain, the expression of *RaPHK* was similar to *RaPGK* (Fig. 3), but increased 2.2-fold in transformed PTAPK4, indicating the integration of one more gene copy. In the transformed strain the heterologous *BsPta* was expressed at the same level than *RaPGK* (Fig. 3), indicating that the genetic locus did not affect the strength of the *RaPGK* promoter.

### Decoupled lipid production from nitrogen starvation

Subsequently, the best performing strain PTAPK4, was cultivated in flask under not limiting nitrogen concentration on glucose and on xylose containing media (C/N 8) to compare its growth performance with the untransformed *ura5* strain (control strain). On glucose, the transformed strain PTAPK4 grew at the same rate than the control, but with a significantly higher specific glucose consumption rate (Table 1). The specific ammonium consumption rate was the same in both strains, as well as their biomass yields. On xylose, the control strain showed a lower growth rate as well as a lower biomass yield than on glucose, reflecting a lower assimilation of this pentose sugar. These observations are in agreement with those recently reported in a wild type strain of *R. toruloides* [29]. On the other hand, the transformed strain PTAPK4, even though grew and consumed xylose and ammonium at the same rate than the control, however it exhibited a higher biomass yield (Table 1). This could indicate an improved efficiency in xylose utilization due to the manipulation of xylulose-5-P flux caused by the overexpression of native Xfpk and heterologous Pta. In a proteomic study carried out in *R. toruloides*, no significant differences were observed in phosphoketolase levels on glucose and on xylose, making not possible to establish the role played by this pathway on xylose utilization [29].

**Table 1:** Growth parameters of parental *ura5* strain and PTAPK4 transformant in shake-flask cultures

	Glucose		Xylose	
	*ura5*	PTAPK4	*ura5*	PTAPK4
μmax	0.27 ± 0.01	0.27 ± 0.03	0.11 ± 0.001	0.12 ± 0.005
q glucose (mmol glucose/g d. w./h)	3.40 ± 0.09	5.03 ± 0.16	N.D	N.D
q xylose (mmol xylose/g d. w./h)	N.D	N.D	1.70 ± 0.20	1.61 ± 0.15
q ammonia (mmol ammonia/g d. w./h)	1.12 ± 0.02	1.10 ± 0.03	0.75 ± 0.03	0.75 ± 0.02
Y biomass (g d. w./g sugar)	0.34 ± 0.004	0.33 ± 0.005	0.25 ± 0.014	0.30 ± 0.015

The cultivation on media at different C/N ratios allowed us to study more specifically the effects of enzymes’ overexpression on decoupling lipid production from nitrogen limitation.

In oleaginous yeasts, cytoplasmic AcCoA is mainly produced from citrate by the activity of ACL, and nitrogen starvation is known to trigger citrate accumulation [7]. Media containing high C/N ratio are then those favoring lipid synthesis. On medium at low C/N ratio (B20), after 65 h of cultivation nitrogen is still present in all the cultures (Table 2) and, as consequence, the lipid content (% of dry weight) was low in *ura5* as well as in wild type strain (16.7% and 21.6% respectively, Table 2). However, PTAPK4 strain showed a higher lipid content (28.7%). Taking in consideration the lipid titer, it was highest in wild type strain. This was the consequence of the highest lipid-free biomass reached by this strain (Table 2). The observation that a lower lipid titer was obtained by PTAPK4 strain, lead to exclude the possibility of a negative effect played on biomass production by uracil content of the medium, as could be supposed for the *ura5* cultivation.

**Table 2:** Lipid and biomass production in shake flasks at different C/N ratios

	48 h					65 h				
	d. w. (g/l)	Lipid titer (g/l)	Lipid content (g/l)	Residual ammonium (g/l)	Residual glucose (g/l)	d. w. (g/l)	Lipid titer (g/l)	Lipid content (g/l)	Residual ammonium (g/l)	Residual glucose (g/l)
WT(RGRDP3)										
B75	7.15 ± 0.46	2.23 ± 0.36	31.2 ± 1.90	0	32.5 ± 0.25	12.9 ± 0.52	6.14 ± 0.62	52.9 ± 2.61	0	14.8 ± 1.01
B37	7.65 ± 0.51	1.38 ± 0.45	18.1 ± 2.31	0.22 ± 0.09	31.6 ± 0.31	12.0 ± 0.43	3.45 ± 0.41	29.1 ± 1.94	0.11 ± 0.06	15.0 ± 1.12
B20	7.57 ± 0.32	1.01 ± 0.21	15.3 ± 2.68	0.69 ± 0.08	32.9 ± 0.21	12.8 ± 0.66	3.31 ± 0.37	21.6 ± 1.98	0.55 ± 0.04	13.2 ± 0.95
*ura5*										
B75	5.27 ± 0.40	1.76 ± 0.36	33.7 ± 3.95	0	37.4 ± 0.11	5.98 ± 1.02	3.10 ± 0.57	51.8 ± 4.31	0	8.52 ± 0.18
B37	5.70 ± 0.34	0.61 ± 0.17	10.7 ± 3.51	0.34 ± 0.03	36.3 ± 0.13	6.40 ± 0.14	1.34 ± 0.35	20.9 ± 2.45	0.08 ± 0.07	10.06 ± 0.29
B20	5.88 ± 0.42	0.63 ± 0.37	10.6 ± 2.57	0.88 ± 0.04	39.4 ± 0.20	6.41 ± 0.39	1.07 ± 0.18	16.7 ± 2.34	0.73 ± 0.07	8.76 ± 0.15
PTAPK4										
B75	6.20 ± 0.27	2.46 ± 0.41	39.7 ± 3.40	0	38.8 ± 0.19	11.8 ± 0.34	8.82 ± 0.26	69.4 ± 2.32	0	3.04 ± 0.17
B37	5.55 ± 0.38	1.66 ± 0.47	29.9 ± 2.87	0.29 ± 0.04	33.3 ± 0.12	8.70 ± 0.19	3.70 ± 0.58	42.5 ± 3.97	0.07 ± 0.05	5.91 ± 0.15
B20	6.72 ± 0.41	1.43 ± 0.34	21.2 ± 2.99	0.82 ± 0.02	38.1 ± 0.15	8.17 ± 0.09	2.35 ± 0.34	28.7 ± 1.42	0.68 ± 0.03	6.17 ± 0.21

PTAPK4 strain showed higher lipid content in comparison to the control strains also on medium at higher C/N ratio (B37), again in presence of residual nitrogen (Table 2). These results clearly indicate that the engineered strain is able to accumulate more lipids than the control strains on media at initial low C/N ratio, when nitrogen is still present in the medium, conditions that are not supportive of lipid synthesis.

**Fig. 4:**
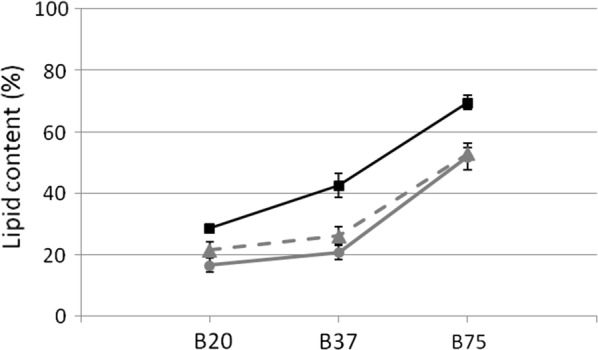
Lipid content (% on dry weight) after 65 h of cultivation at different C/N ratios (filled up pointing triangle, wild-type RGRDP3 strain; filled circle ura5 strain; filled square, PTAPK4 strain)

As expected, in all the strains lipid accumulation was higher in medium at C/N ratio 75 (B75), in which nitrogen has been completely depleted already after 48 h of cultivation, and then causing the activation of the mechanisms triggering cytoplasmic citrate accumulation (Table 2). This indicates that the mechanisms regulating citrate accumulation are operating in all the strains, causing a direct correlation between lipid accumulation and C/N ratio. Anyway, we found that the lipid level reached by the PTAPK4 strain was higher than the control levels at all the C/N ratios analysed (Fig. 4).

In conclusion, the results from flask cultivations indicate that the overexpression of homologous Xfpk and heterologous Pta exerts a positive role on xylose utilization in *R. azoricum*. The engineered strain exhibits in fact improved xylose utilization (Table 1). Furthermore, the engineered strain reaches higher lipid content even in presence of nitrogen, demonstrating that the introduction of the new pathway supplying cytosolic AcCoA makes the strain less dependent on citrate accumulation, and then less sensitive to the mechanisms linked to nitrogen limitation (Table 2). This effect is similar to the one obtained in *Y. lipolytica* upon overexpression of heterologous Xfpk and Pta [17].

### Lipid production in fed-batch process

**Fig. 5:**
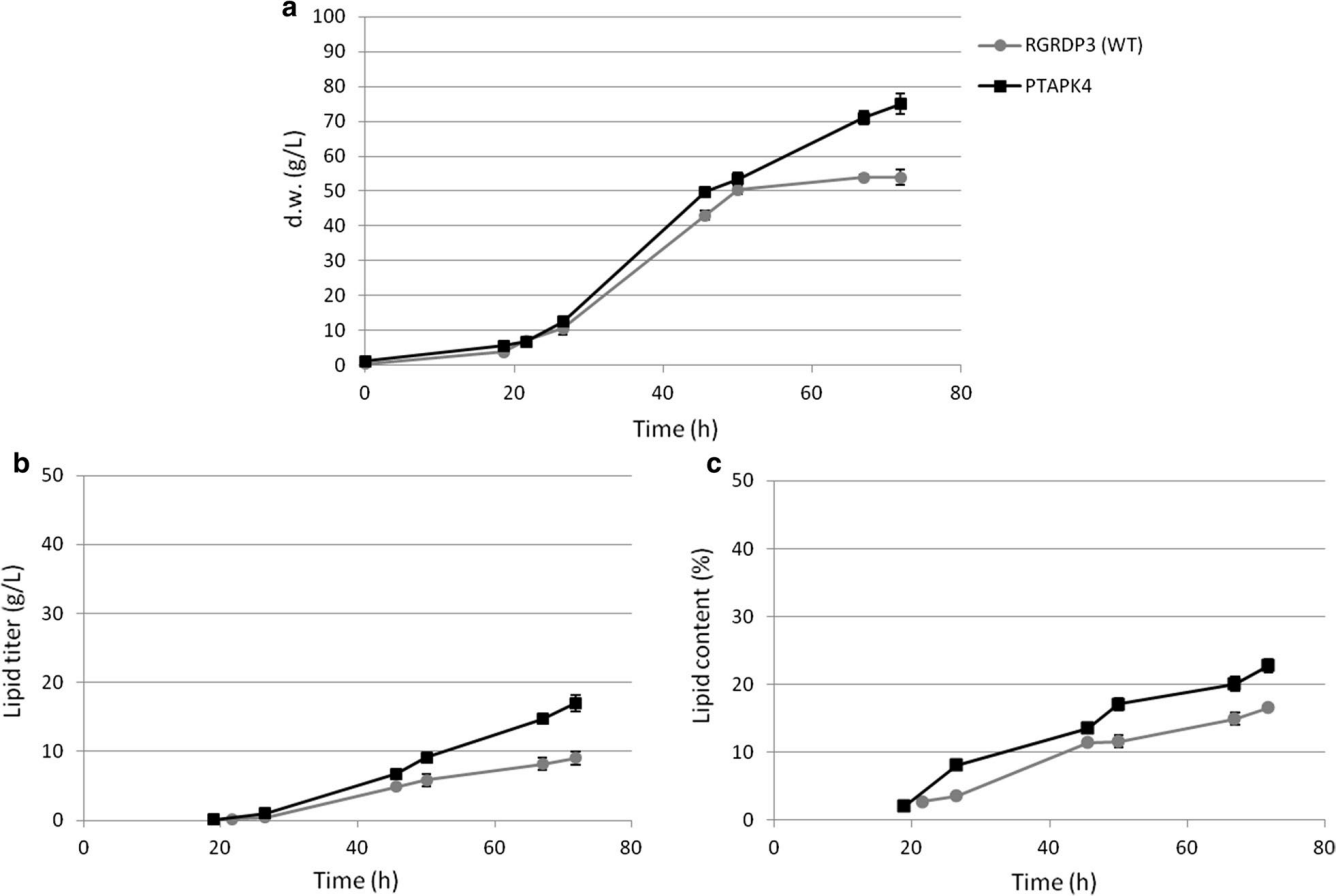
Fed-batch cultures by PTAPK4 strain (filled square, black line) compared to the wild type (filled circle, grey line). **a** Kinetic of dry weight (g/l), **b** lipid titer (g/l), **c** lipid content (% on dry weight)

In order to analyze under well-controlled conditions of pH and oxygen supply the positive effects on lipid production of engineered AcCoA pathway observed in flask, the transformed strain PTAPK4 was cultivated in bioreactor. In addition, in these experiments we decided to simulate industrial conditions. For this reason, we cultivated as control strain only the wild type, which is usually employed for industrial purpose. We utilized as carbon source a glucose–xylose mixture (2:1), typical of a lignocellulosic hydrolysate. During the first part of the fed-batch process (50 h), a feeding of sugars and nitrogen was ran for both strains, as usually done, to obtain high cellular biomass concentration, which correspond to cultivation at low C/N ratio. This condition repress cytoplasmic AcCoA synthesis by ACL, and then lipid accumulation by this pathway. Under these conditions, after 50 h the wild type strain reached a biomass concentration of 50.3 g/l and, as expected, a low lipid content (11.6%), resulting in a low lipid concentration (5.8 g/l). However, under the same conditions, the biomass produced by the PTAPK4 strain contained more lipids (17.1%). As consequence, since the transformed strain produced the same amount of lipid-free biomass than the wild type (44 g/l for both strains), a 50% higher lipid concentration (9 g/l) was obtained by PTAPK4 (Fig. 5). This confirmed that in our engineered strain the overexpression of the homologous Xfpk in addition to the heterologous Pta, creating a new source of cytoplasmic AcCoA, was responsible of improvement in lipid production even in presence of nitrogen. In addition, this result demonstrates that the lower level of lipid-free biomass obtained in flask cultivation by PTAPK4, as well as by *ura5* mutant, was probably due to a greater oxygen demand from these strains, which in turn can represent an effect resulting from the mutagenesis treatment. This effect is overcame by cultivation in well-aerated condition in bioreactor. This is in contrast to the results reported in *R. toruloides*, in which the strain expressing the heterologous Pta cultivated in bioreactor showed a lipid content 10% lower than the control strain, but the process resulted in 7.6% increased final lipid concentration due to the higher level of biomass obtained [24]. We cannot nevertheless exclude that the differences are due also to different cultural conditions utilized in our study.

After 50 h of cultivation under the same conditions for the two strains (wild type and transformed), we decided to change the feeding strategy, in order to induce lipogenesis, as normally operated during an industrial process with wild type strain. In the wild type cultivation the nitrogen feeding was then stopped, causing nitrogen depletion. After further 22 h, the biomass increased its lipid content, as expected, and consequently the final lipid concentration reached in this process was 9 g/l (Fig. 5). On the contrary, in the process performed by PTAPK4 strain, we decided to extend the nitrogen feeding to allow continuing the cellular biomass synthesis, and, due to the positive effect exerted by the presence of the new AcCoA pathway on lipid accumulation even in presence of nitrogen, in order to evaluate the possibility to obtain a higher lipid concentration. Under this condition, we achieved an increase of 89% in comparison to the wild type strain, reaching a final lipid concentration of 17 g/l (Fig. 5). This reflected then the ability of the transformed strain to produce new cellular biomass and, although under not lipogenic conditions, with a higher lipid content than the one obtained by the wild type under lipogenic condition (22.7% and 16.6% respectively). The comparison of lipid yields furthermore evidenced the advantage of the transformed strain over the wild type in performing this process, being 0.13 and 0.05, respectively.

## Conclusions

Our work demonstrates that we decoupled lipogenesis from nitrogen starvation in *R. azoricum* by overexpressing homologous Xfpk and by introducing heterologous Pta encoding genes. By simulating an industrial process, the engineered strain achieved an increase of 89% in final lipid concentration in comparison to the wild type strain. Noteworthy, this goal was reached without negatively affecting cellular biomass production, and improving xylose utilization. In conclusion, this metabolic modification allows to efficiently improving lipid production in cultural conditions that can be suitable for the development of industrial bioprocesses using lignocellulosic hydrolysates.

## Supplementary information

**Additional file 1: Table S1.** Primer sequences employed for the assembly of pTU-PT-PTAPK vector, for the screening of mutants and for the RT-qPCR experiments. **Fig. S1.** Comparison of nucleotide sequence (A) and amino acid sequence (B) of *URA5* between wild-type RGRDP3 and *ura5* strain. **Fig. S2.** Agarose gel of PCR products generated by using BsPta and RaPHK specific primers on clones obtained by transformation of *ura5* strain with pTU-PT-PTAPK. **Fig. S3.** Lipid titer (g/l) after 65 h of cultivation of the seven clones obtained by transformation of *ura5* strain with pTU-PT-PTAPK, and showing the insertion of both genes. **Fig. S4.** Time course of residual glucose (g/l) (●, black line), residual xylose (g/l) (■, black line), residual ammonium (g/l) (▲, gray line) during fed-batch culture of wild-type RGRDP3 (A) and PTAPK4 strain (B).

## Data Availability

The datasets supporting the conclusions of this article are included within the article and its additional files.
