# Supplementary material for: Engineering cytoplasmic acetyl-CoA synthesis decouples lipid production from nitrogen starvation in the oleaginous yeast Rhodosporidium azoricum
Source: Microb Cell Fact. 2019 Nov 14;18:199. doi: 10.1186/s12934-019-1250-6 (PMC6854766; doi:10.1186/s12934-019-1250-6)
Supplement: Supplementary file 1 — Additional file 1: Table S1. Primer sequences employed for the assembly of pTU-PT-PTAPK vector, for the screening of mutants and for the RT-qPCR experiments. Fig. S1. Comparison of nucleotide sequence (A) and amino acid sequence (B) of URA5 between wild-type RGRDP3 and ura5 strain. Fig. S2. Agarose gel of PCR products generated by using BsPta and RaPHK specific primers on clones obtained by transformation of ura5 strain with pTU-PT-PTAPK. Fig. S3. Lipid titer (g/l) after 65 h of cultivation of the seven clones obtained by transformation of ura5 strain with pTU-PT-PTAPK, and showing the insertion of both genes. Fig. S4. Time course of residual glucose (g/l) (●, black line), residual xylose (g/l) (■, black line), residual ammonium (g/l) (▲, gray line) during fed-batch culture of wild-type RGRDP3 (A) and PTAPK4 strain (B). [file 12934_2019_1250_MOESM1_ESM.pptx]

## Slide 1
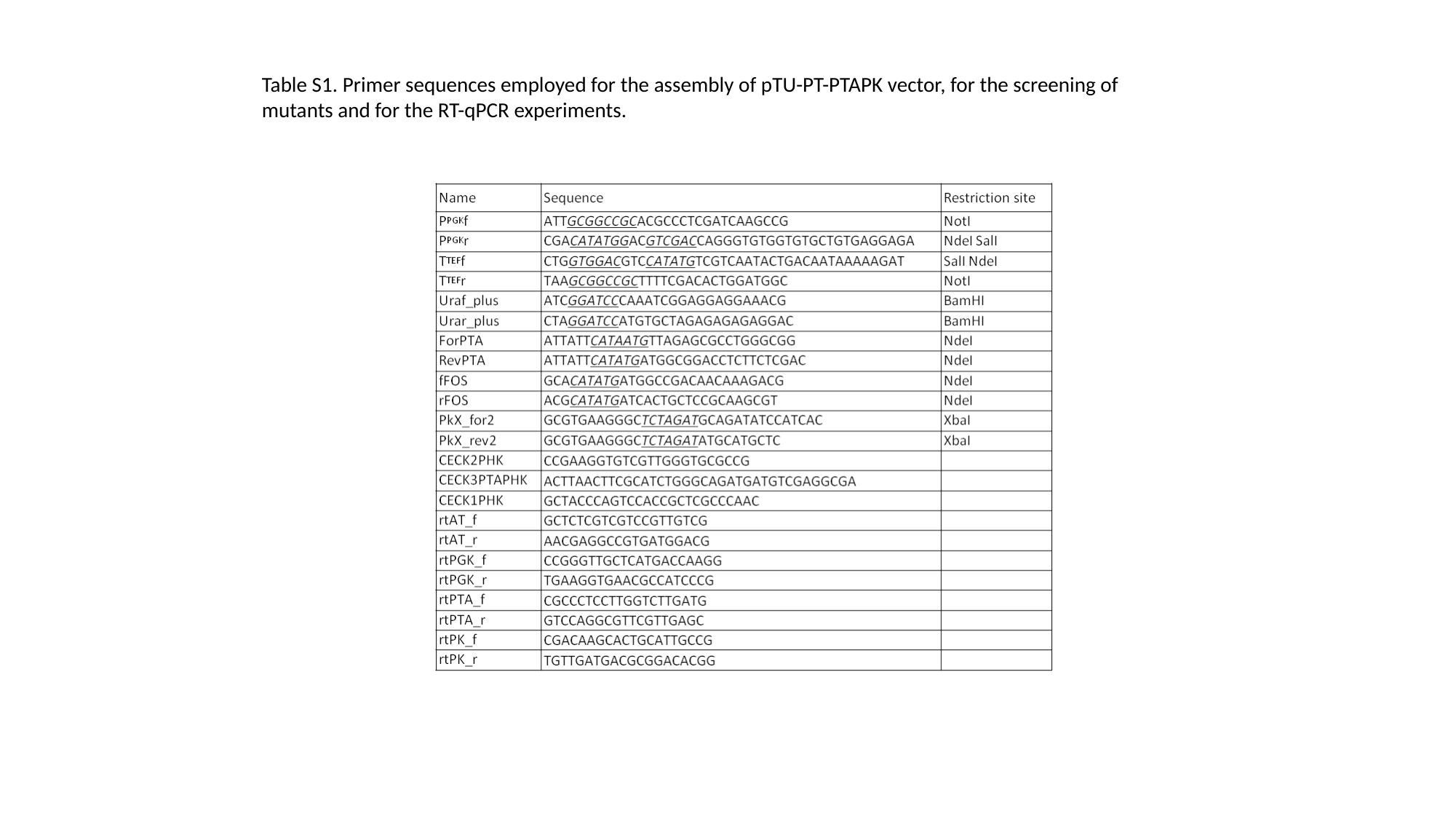

Table S1. Primer sequences employed for the assembly of pTU-PT-PTAPK vector, for the screening of mutants and for the RT-qPCR experiments.

## Slide 2
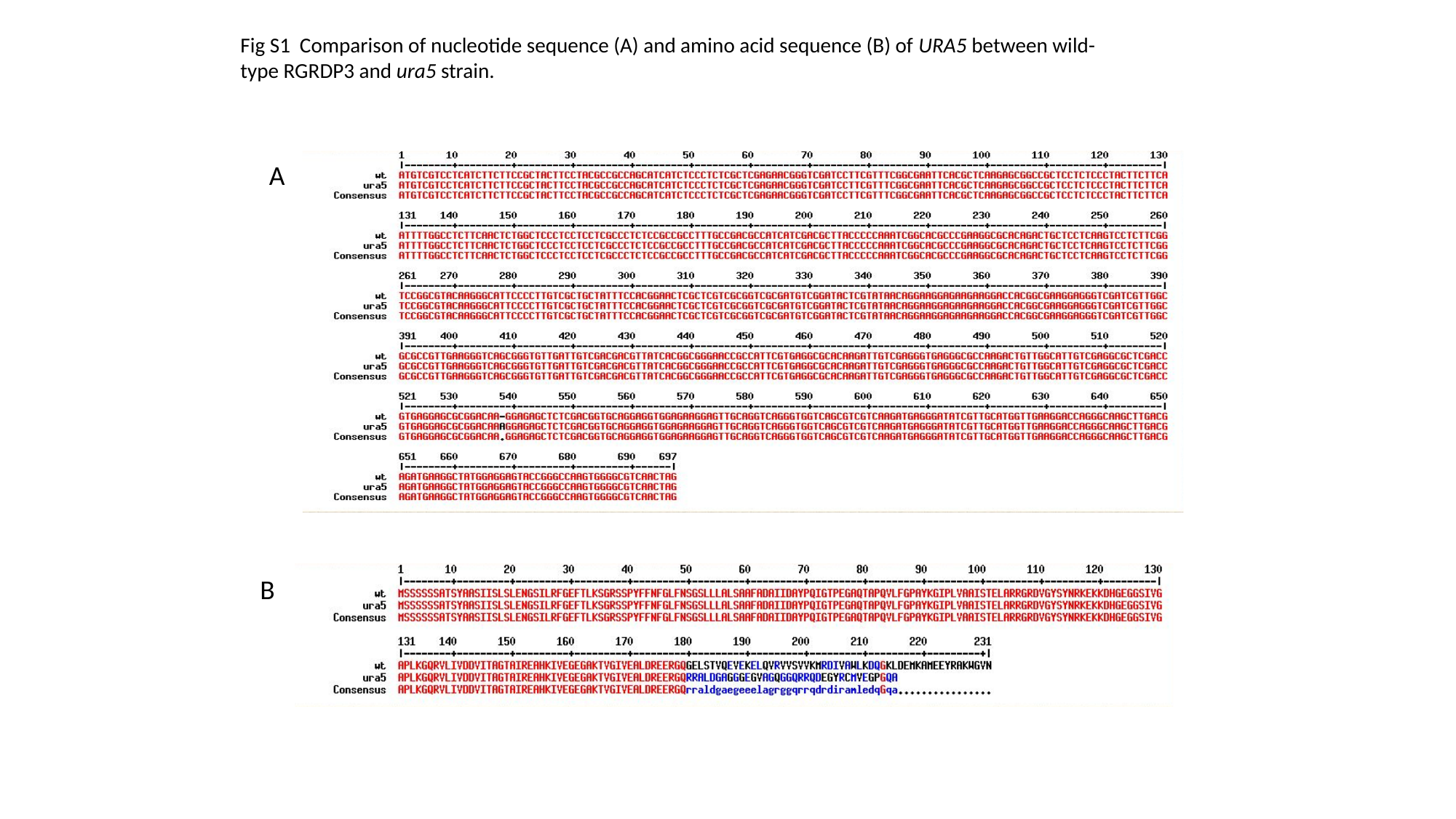

Fig S1 Comparison of nucleotide sequence (A) and amino acid sequence (B) of URA5 between wild-type RGRDP3 and ura5 strain.
A
B

## Slide 3
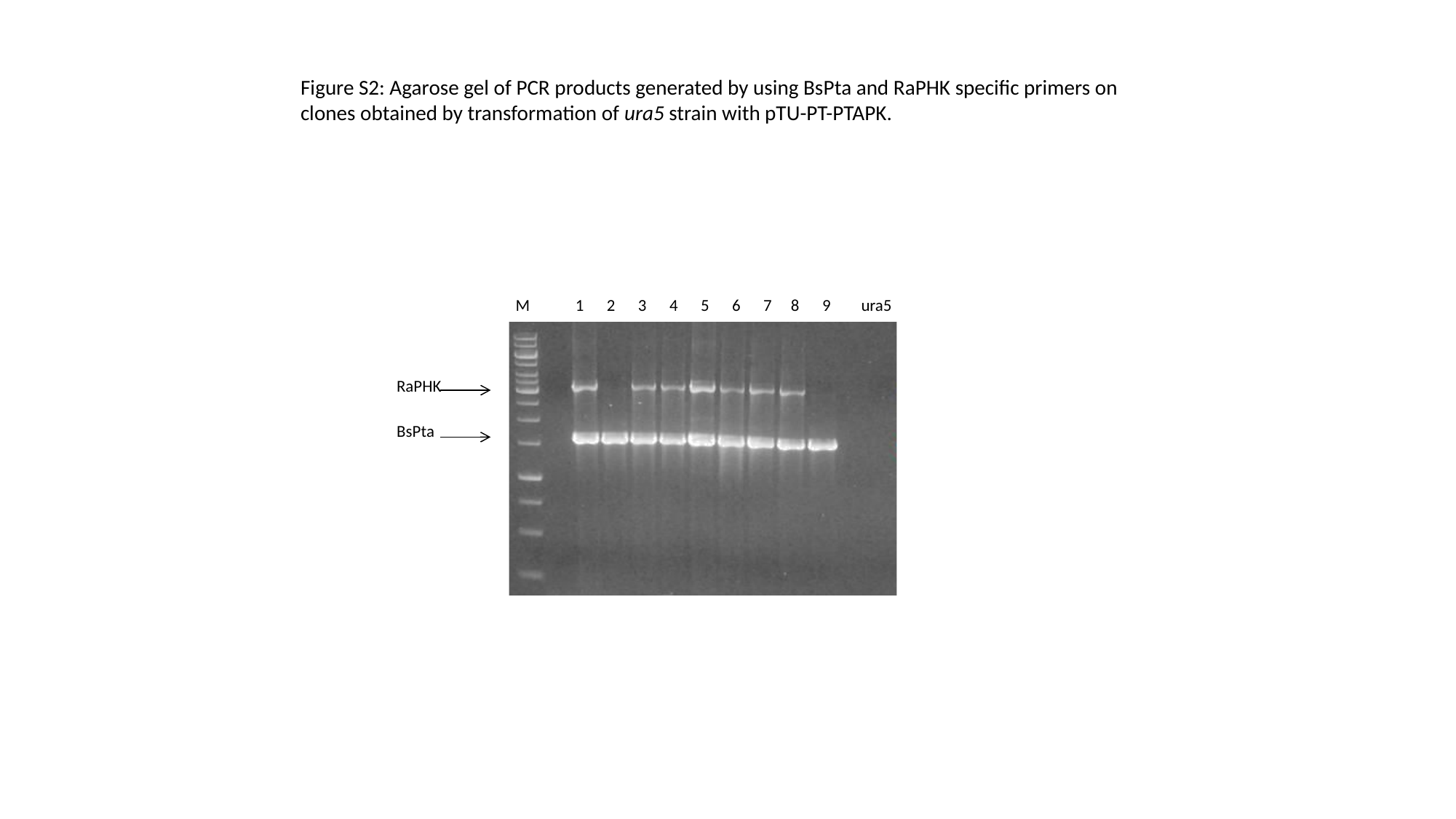

Figure S2: Agarose gel of PCR products generated by using BsPta and RaPHK specific primers on clones obtained by transformation of ura5 strain with pTU-PT-PTAPK.
M 1 2 3 4 5 6 7 8 9 ura5
RaPHK
BsPta

## Slide 4
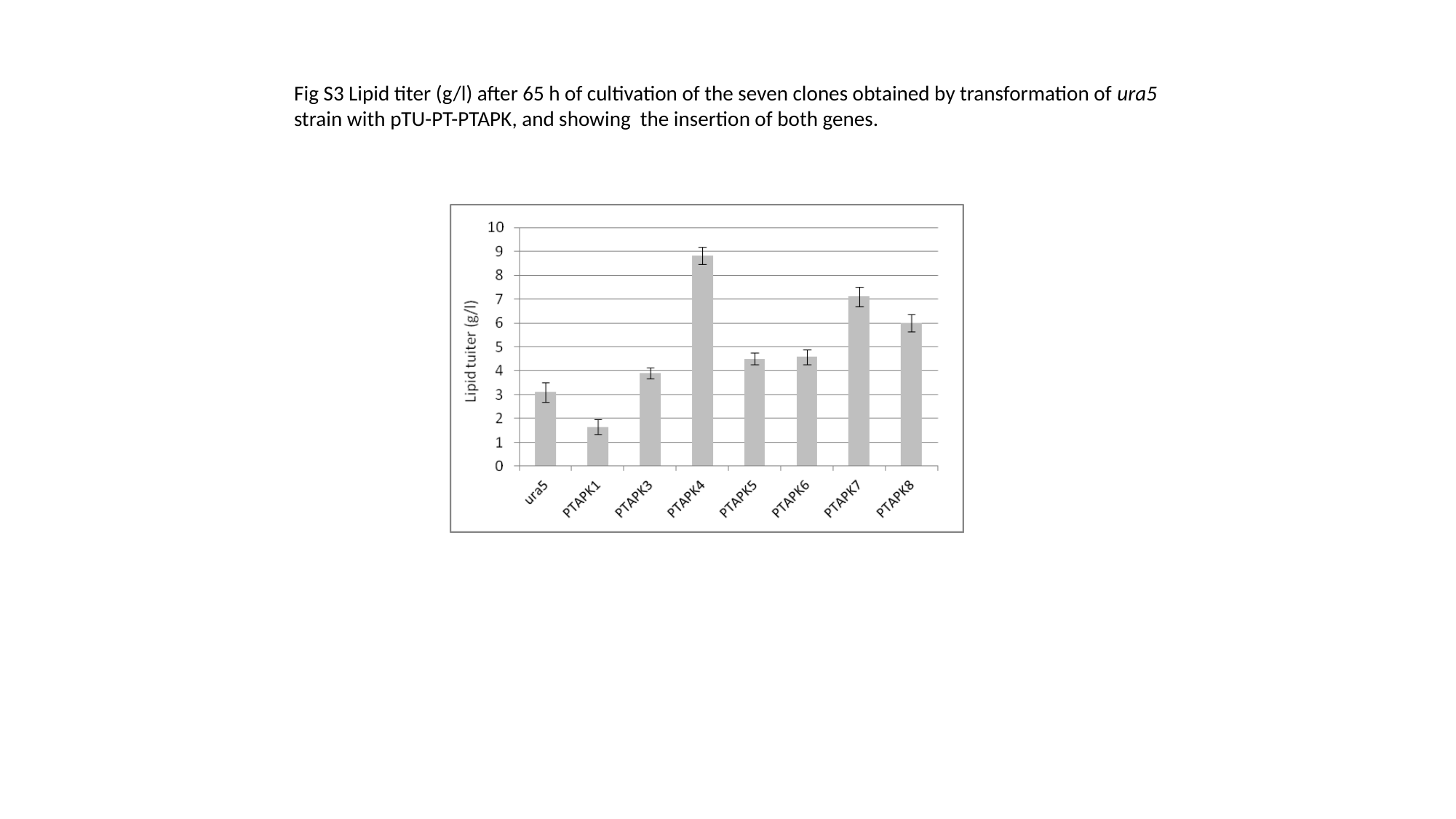

Fig S3 Lipid titer (g/l) after 65 h of cultivation of the seven clones obtained by transformation of ura5 strain with pTU-PT-PTAPK, and showing the insertion of both genes.

## Slide 5
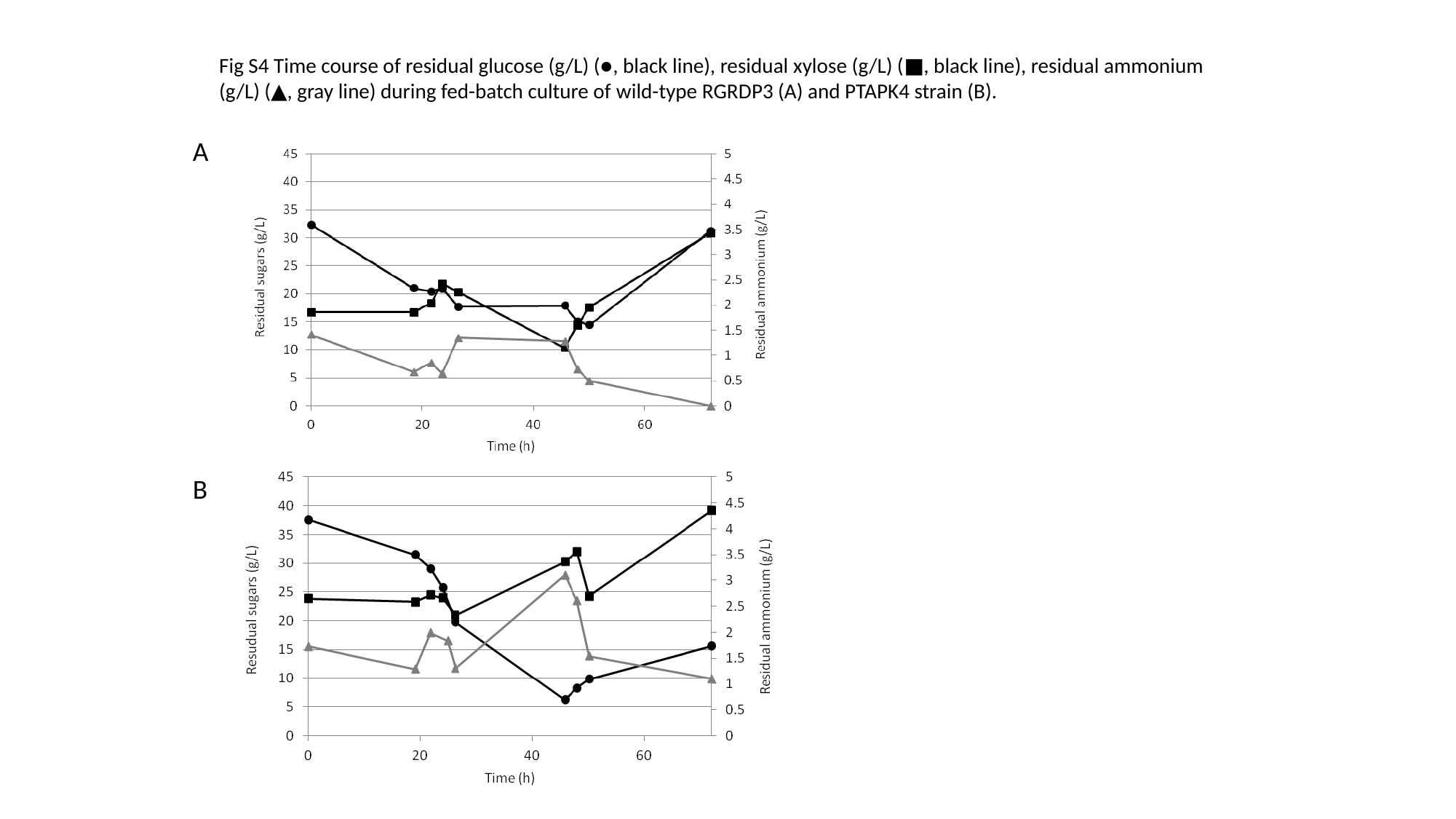

Fig S4 Time course of residual glucose (g/L) (●, black line), residual xylose (g/L) (■, black line), residual ammonium (g/L) (▲, gray line) during fed-batch culture of wild-type RGRDP3 (A) and PTAPK4 strain (B).
A
B
